# Gold complex compounds that inhibit drug-resistant *Staphylococcus aureus* by targeting thioredoxin reductase

**DOI:** 10.3389/frabi.2023.1179354

**Published:** 2023-08-21

**Authors:** Nagendran Tharmalingam, Shi Xu, Lewis Oscar Felix, Biswajit Roy, Ming Xian, Eleftherios Mylonakis, Beth Burgwyn Fuchs

**Affiliations:** ^1^ Division of Infectious Diseases, Warren Alpert Medical School of Brown University, The Miriam Hospital, Providence, RI, United States; ^2^ Department of Chemistry, Brown University, Providence, RI, United States

**Keywords:** antimicrobial compound, auranofin, biofilm, *Staphylococcus aureus*, thioredoxin reductase (TrxR), thiol, gold compounds

## Abstract

**Introduction:**

There is a significant need for new antimicrobial compounds that are effective against drug-resistant microbes. Thioredoxin reductase (TrxR) is critical in redox homeostasis and was identified as a potential drug target and confirmed through inhibition by compounds auranofin and Bay11-7085.

**Methods:**

Additional TrxR inhibitors were designed and found to exhibit antimicrobial activity against Gram-positive (*Enterococcus faecium* and *Staphylococcus aureus*) and glutathione-deficient bacteria (*Helicobacter pylori*). Investigational compounds were tested for antimicrobial activity, anti-biofilm efficacy, target impact, and cytotoxicity.

**Results:**

The first-generation molecules AU1 and AU5 inhibited TrxR activity and inhibited methicillin-resistant *S*. *aureus* strain MW2 with minimal inhibitory concentrations (MIC) of 0.125 and 0.5 μg/mL, respectively. In an *S. aureus* enzymatic assay, AU1 inhibited TrxR enzymatic activity in a dose-dependent manner causing a decrease in intracellular free thiols. In addition, biofilm studies demonstrated that AU1 and AU5 reduced biofilm formation at 1X MIC and disrupted mature biofilms at 4X MIC. Cytotoxicity profiles were created using human cell lines and primary cells with LD_50_ exceeding MICs by at least 12X.

**Discussion:**

Thus, AU1 and AU5 were TrxR inhibitors that yielded low-concentration antimicrobial activity impacting *S. aureus* in planktonic and biofilm forms with limited toxic liability.

## Introduction

With the continued emergence of drug-resistant microbes, there is a significant need to develop new antimicrobial compounds that engage novel or underutilized drug targets. Bacterial thioredoxin reductase (TrxR) was identified as a viable antimicrobial target that is not yet taxed by the standard of care antibiotic arsenal (Lu et al., 2013; Harbut et al., 2015; Gustafsson et al., 2016; Ruth et al., 2019; Felix et al., 2021). As an antioxidant, the thioredoxin system plays an important role in Gram-positive bacteria (Scharf et al., 1998; Lu et al., 2013; Harbut et al., 2015). The thioredoxin system is functionally redundant with the glutathione system found in most Gram-negative bacteria, but this is inherently missing in Gram-positive bacteria, making it essential to this class of microbes (Lu and Holmgren, 2014).

The thioredoxin system is comprised of NADPH, thioredoxin reductase (TrxR), and thioredoxin (Trx) (**Supplemental Figure 1A**), which acts as an antioxidant by facilitating the reduction of reactive oxygen species via the transfer of electrons from the NADPH donor. TrxR is a catalyst within the system, transferring electrons from NADPH to Trx (Lu and Holmgren, 2014). Inhibition of TrxR results in ROS accumulation, leading to apoptosis, leaving GSH-negative cells susceptible to oxidative stress (Duan et al., 2014; Hwang-bo et al., 2017).

A high throughput screen (HTS) in a *Caenorhabditis elegans* infection model identified anti-*Staphylococcus aureus* compounds that included auranofin. Auranofin has emerged as a control compound that inhibits TrxR and exerts antimicrobial activity against Gram-positive bacteria (Harbut et al., 2015; Roder and Thomson, 2015; Hwang-bo et al., 2017; May et al., 2018). As, an FDA-approved compound, auranofin is used to treat rheumatoid arthritis but is known to have off-target effects (Kean et al., 1997). Using auranofin as a proof-of-concept compound, additional small molecules were explored to develop new TrxR inhibitors specific to a Gram-positive TrxR. In the nematode infection model, auranofin prolonged the survival of *C*. *elegans* nematodes infected with *S. aureus* (Fuchs et al., 2016) and an evaluation of over 500 clinical isolates found that auranofin demonstrated consistent anti-*S*. *aureus* activity with minimal inhibitory concentrations (MIC) ranging from 0.125 to 1 μg/mL. Among the tested strains were methicillin-resistant *S*. *aureus* (MRSA), methicillin-susceptible *S*. *aureus* (MSSA), and vancomycin-intermediate *S*. *aureus* (VISA) isolates (Tharmalingam et al., 2019). Auranofin sensitivity for the diverse *S*. *aureus* collection suggests that known drug-resistance mechanisms are not deployed against auranofin. *S*. *aureus* was also exposed to auranofin over a 25-day period, a time frame that captures most antibiotic treatment regimens. Extended auranofin exposure did not alter the *S*. *aureus* auranofin susceptibility profile (Tharmalingam et al., 2019). Thus, auranofin resistance did not occur, even in drug-resistant isolates.

Harbut et al. also demonstrated that auranofin is effective against *S*. *aureus* and non-replicating *Mycobacterium tuberculosis* (MTB), a bacterium that does not fit within the Gram system but, like *S*. *aureus*, is Trx-dependent and GSH-independent (Harbut et al., 2015). Further, *in vivo* analysis using a murine peritonitis infection model demonstrated that auranofin treatment prolonged survival (Harbut et al., 2015). The authors concluded that auranofin is a potent inhibitor of bacterial TrxR, resulting in redox disruption that reduces free thiols and bacterial death (Harbut et al., 2015).

Using auranofin and another HTS hit as a model compounds, we developed two phosphorus-gold-sulfur compounds that inhibit the Gram-positive bacterium *S*. *aureus*, the causative agent of infections that range from soft tissue infections to systemic bacteremia (Guo et al., 2020). The two lead compounds caused bacteria growth reduction and even killed bacteria by targeting *S*. *aureus* TrxR enzymatic activity with low mammalian cell cytotoxicity.

## Materials and methods

### TrxB protein expression vector

We previously amplified the *trxB* sequence, which codes for TrxR, from *S*. *aureus* MW2 using primers: TrxB-F-ATGACTGAAATAGATTTTGATATAGCAATTATCGGTGC and TrxB-R-TTAAGCTTGATCGTTTAAATGTTCAATATATTCCGC and cloned it into the pET30a plasmid (Millipore Sigma, St. Louis, MO, USA)., and confirmed with sequencing (Tharmalingam et al., 2019). The plasmid was isolated and transferred into *E. coli* BL21 for expression and purification of TrxB (TrxR).

### TrxB enzymatic assay screen

To accomplish the subscreen and identify *S*. *aureus* TrxR specific inhibitors, *S. aureus* MW2 TrxR was cloned into pET30a, expressed in *E. coli* (BL21), and the purified protein was isolated and used for the TrxR enzymatic sub-screening assay. The subscreen consisted of an enzymatic assay to define additional TrxR inhibitors by measuring the conversion of 5,5’-dithio-*bis*(2-dinitrobenzoic acid) (DTNB) to 5-thio-2-nitrobenzoic acid (TNB), which has a colorimetric readout, using the Cayman Chemical Thioredoxin reductase Colorimetric Assay Kit with some modifications. *S*. *aureus* TrxR was used to convert DTNB to TNB (**Supplemental Figure 1B**) with incubation periods of 10 minutes or greater achieving significance with a paired t-test (*P*<0.05). Measurable activity reduction was achieved with auranofin (5 μg/mL) compared to vancomycin (10 μg/mL) (*P*<0.01, using an unpaired t-test) (**Supplemental Figure 1C**). TrxR specific inhibitory activity was evaluated using 40 μL of *S*. *aureus* TrxR enzyme (0.5 μg/μL) per 200 mL of the reaction mixture for 15 minutes, comparing auranofin to other sulfur-containing compounds (all tested at 10 μg/mL), including known anti-*S*. *aureus* compounds. Indeed, TrxR inhibitory activity was specific to auranofin and did not occur with the other standard of care antibiotics (**Supplemental Figure 1D**). Within the screen, auranofin was used as a positive control for the assay and DMSO served as the negative control, achieving a Z-factor of 0.72 for the screening assay. From the compound panel, small molecules that significantly inhibit TrxR in the reduction of DTNB to TNB were considered hits (**Supplemental Figure 1E**).

For the screening assay, diluted TrxR assay buffer (Cayman Chemical, Ann Arbor, MI) (100 μL) was mixed with the investigational compounds (20 μL) in a 96 well-plate format and combined with *Sa*-TrxB (40 μL; 0.5 μg/mL), DTNB (20 μL) and NADPH (20 μL). The reaction entailed combining NADPH and Sa-TrxB with DTNB, which was converted to TNB, producing a yellow color. The inclusion of *Sa*-TrxB enzyme inhibitors (provided in the kit) in the assay resulted in no or reduced color development. Reaction test plates were incubated in the dark at 37 °C for 30 min and subsequently read at 420 nm using a Vmax microplate reader (Molecular Device, Sunnyvale, CA, USA.).

The assay was applied to 169 small molecules (ICCB, Harvard Medical School, Boston MA, USA) identified as hits in a previous HTS of antibacterial compounds that exhibited anti-*S*. *aureus* activity (Rajamuthiah et al., 2014; Fuchs et al., 2016; Kim et al., 2018) and were thus, interrogated in a subscreen for TrxR inhibitors in duplicate. Using auranofin as a positive control and DMSO as a negative control, a Z’-factor of 0.7 was calculated to assess the robustness of the subscreen process using the TrxR enzymatic assay, establishing an effective screen. Upon screening the 169 compound anti-*S*. *aureus* collection, small molecules that inhibited the TrxR enzymatic assay two standard deviations below the DMSO control, which was defined as OD_420_ 0.24, were designated as hits to follow further.

### AU1 and AU5 synthesis

A solution of 1,8-diazabicyclo(5.4.0)undec-7-ene (DBU) (1.2 mmol) in 1 mL dichloromethane (DCM) was dropped into a solution of solution of chloro(triethylphosphine)gold(I) (1.0 mmol) and 4-tert-butylbenzenethiol (1.2 mmol) in 5 mL DCM. The mixture was stirred at 360 rpm at room temperature for 2 h. The mixture was then concentrated and subjected to column chromatography (30% ethyl acetate/hexane) to give AU1 (331 mg, 69% yield). NMR spectra were recorded for ^1^H NMR (400 MHz, CDCl_3_) δ 7.53 – 7.41 (d, *J* = 8.3 Hz, 2H), 7.17 – 7.08 (d, *J* = 8.3 Hz, 2H), 1.86 (q, 6H), 1.31 – 1.12 (m, 18H);^13^CNMR (100MHz, CDCl_3_) δ 146.4, 137.4, 132.2, 125.9, 34.1, 31.4, 18.2, 9.0; ^31^PNMR (162 MHz, CDCl_3_) δ 37.21 (**Scheme 1**).

To a suspension of chloro(triphenylphosphine) gold(I) (0.5 mmol) in tetrahydrofuran (THF) 10 mL of 4-tert-butylbenzenesulfinato silver(I) (0.5 mmol) was added and stirred in the dark at 360 rpm for 2 h. The mixture was then centrifuged (25 °C, 10,500 rpm for 15 min) and the supernatant was concentrated. Ether (100 mL) was then added, and the mixture was stirred for at 360 rpm 1 h. The crude product was collected by filtration and reprecipitated by THF/pentane to get AU5 (229 mg, 70% yield). NMR spectra were recorded for ^1^H NMR (400 MHz, CDCl_3_) δ 7.95 (d, *J* = 8.4 Hz, 2H), 7.76 – 7.39 (m, 17H), 1.34 (s, 9H); ^13^CNMR (100MHz, CDCl_3_) δ 146.1, 134.2, 134.1, 132.2, 129.4, 129.3, 126.3, 125.4, 34.2, 31.2; ^31^PNMR (162 MHz, *d6*-DMSO) δ 42.81 (**Scheme 2**). 
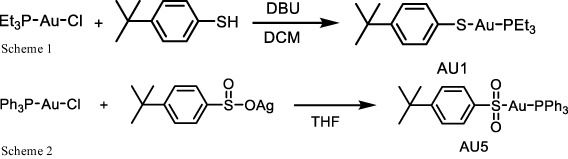


### Antimicrobial susceptibility assays

*In vitro* antibiotic susceptibilities were determined using the broth microdilution assay established by the Clinical and Laboratory Standards Institute (Clinical and Laboratory Standards Institute, 2012). Broth microdilution assays were carried out with 2 biological replicates, each in triplicate, Mueller–Hinton broth (MHB; BD Biosciences, Franklin Lakes, NJ, USA) with a total assay volume of 100 μL in 96-well plates (BD Biosciences, Franklin Lakes, NJ, USA). Two-fold serial dilutions of compounds were prepared at a range of 0.01– 64 μg/mL in 50 μL of MHB. The initial bacterial inoculum was adjusted to OD_600_ 0.06, 50 μL was, added to the test compounds, and then incubated at 35°C for 18 h. Post incubation, OD_600_ was measured using a Vmax microplate reader (Molecular Devices, San Jose, CA), and the lowest concentration of test compound that suppressed bacterial growth was reported as the MIC. Broth culture from the MIC assay (10 μL) was plated on Mueller–Hinton agar (BD Biosciences, Franklin Lakes, NJ, USA), and colony-forming units (CFUs) were enumerated after overnight incubation at 37°C. The lowest concentration of compound that resulted in no growth was reported as the minimal bactericidal concentration (MBC).

### Diamide assay

An overnight culture of *S*. *aureus* MW2 was inoculated in tryptic soy broth (TSB) media and grown with agitation. The cells were centrifuged, washed with PBS, and diluted to an OD_600_ 0.4 (10^8^ CFU/mL) in PBS. The cells were then treated with auranofin (0.5 µg/mL), AU1 (0.5 µg/mL), AU5 (0.5 µg/mL), and diamide (5 mM and 10 mM) alone or in combinations for 3 h at 37°C. The cells were then serially diluted and plated on tryptic soy agar (TSA) media and incubated at 37°C for 24 h. Colonies that grew on plates were enumerated to determine the effect of the drug and diamide on *S. aureus*.

### Thiol depletion assay

*S. aureus* MW2 was treated for 15 min with the indicated (0.125, 0.25, 0.5, or 0.75 µg/mL) concentrations of auranofin, AU1, AU5, or ampicillin. After treatment bacteria were washed twice in PBS and then resuspended in 100 mM potassium phosphate, monobasic, pH 7.4, containing 1 mM ethylenediaminetetraacetic acid to promote lysis. Cells were incubated in the lysis solution for 30 min at 60°C and then vortexed briefly. The solution was centrifuged at 13,000 g for 10 min to collect cell debris. Lysate was collected and 50 μL was transferred to a 96-well plate. Thiols were quantified using the Fluorometric Thiol assay kit following the manufacturer’s instructions (Millipore Sigma, St. Louis, MO, USA). The fluorescence intensity was measured after a 30 min incubation time at λ_ex_ = 490/λ_em_ = 535 nm using a Vmax plate reader.

### Time to kill assays

*S*. *aureus* MW2, a methicillin-resistant *S*. *aureus* (MRSA) isolate, was used to determine the bacteriostatic/bactericidal properties of test compounds, as previously described (Rajamuthiah et al., 2015). Assays were carried out as 2 biological replicates with n=3 for each in 24 well plates (BD Biosciences, Franklin Lakes, NJ, USA). Briefly, *S. aureus* MW2 cells were streaked on TSB agar from a frozen glycerol stock, and after overnight incubation, a single colony was inoculated on 2 mL of TSB and incubated at 37°C with agitation. The overnight culture of *S*. *aureus* MW2 was centrifuged at 4000 g for 5 min and then washed twice with PBS and collected through centrifuged at 4000 g for 5 min. The PBS was discarded and cells were suspended in TSB and aliquoted into a 24-well plate at a density of 10^8^ cells/mL as a 1 mL volume. Test compounds were added such that the final concentration was 4 × MIC, and the cultures were incubated at 37°C with agitation. At designated time intervals, 5 μL aliquots from each test condition were serially diluted in TSB and plated onto TSA (BD Biosciences, Franklin Lakes, NJ, USA). CFUs were enumerated after overnight incubation at 35°C, and data were plotted using GraphPad Prism 9.0 (San Diego, CA, USA) to report the means and standard deviation of growth as measured by CFU/mL.

### Biofilm assessment

*S*. *aureus* MW2 strain was cultured in TSB with glucose for 24 h and cells were suspended at an OD_600_ of 1.0 and further diluted 1:40 fold in 100 μL of MHB (Gwisai et al., 2017). Diluted bacterial cells were added to 100 μL of test compounds (serially diluted 16 to 0 μg/mL) and incubated for 24 h to assess the biofilm formation inhibition. For the disruption of matured biofilm, diluted cells in 100 μL were added to the 96-well plate and incubated for 24 h with no agitation. After 24 h, the plates were washed three times with phosphate buffered saline (PBS: 137mM NaCl, 2.7mM KCl, 4.3mM Na_2_HPO_4_-7H_2_O, 1.47mM KH_2_PO_4_ in a liter of water), and 200 μL of test compounds at designated concentrations (16 to 0 μg/mL) were added to the 96-well plates and incubated to assess the biofilm disruption assay. After appropriate incubation times, the plates used for biofilm inhibition and disruption assay were subjected to 3x wash with PBS (200 μL), and 200 μL of test medium with Alamar using the Alamar Blue High Sensitivity kit reagent (ThermoFisher Scientific, Waltham, MA, USA) to evaluate biofilm after incubated for 4 h, and the optical density was measured at 570 nm using Vmax microplate reader (Molecular Devices San Jose, CA, USA.) and the data were plotted using GraphPad Prism. Biofilm assays were completed in triplicate.

### Immortalized cell line toxicity

A hepatic cell line (HepG2), lung carcinoma cell line (A549), and kidney cell line (HKC) were used to test the cytotoxicity of investigational compounds. Cells were grown in DMEM (ThermoFisher Scientific, Waltham, MA, USA) supplemented with 10% fetal bovine serum (FBS) (ThermoFisher Scientific, Waltham, MA, USA) and 1% penicillin/streptomycin (ThermoFisher Scientific, Waltham, MA, USA) and maintained at 37°C in 5% CO_2_. 2.5x10^4^ cells in 100 μL of DMEM with 10% FBS were added to wells of 96-well plates 24 h prior to the experiments. Compounds were serially diluted in serum, and 100 μL antibiotic-free DMEM was added to the monolayer and incubated at 37°C in 5% CO_2_ for 24 h. At 2 h before the end of the incubation period, 10 μL of 2-(4-iodophenyl)-3-(4-nitrophenyl)-5-(2, 4-disulfophenyl)-2H-tetrazolium (WST-1) solution (Roche, Mannheim, Germany) was added to each well. WST-1 reduction was monitored at 450 nm using a Vmax microplate reader. Assays were performed in triplicate, and the percentage survival was calculated by comparing the compound-treated wells to the DMSO vehicle control wells.

### Hemolysis of human erythrocytes

The hemolytic activity of auranofin, AU1, and AU5 were tested using human erythrocytes (Rockland Immunochemicals, Limerick, PA, USA) (Lin et al., 2020). Cells were washed with PBS and diluted to 4% human red blood cells (hRBCs) with PBS. In a 96-well plate, RBCs were added in a 100 μL volume and then 100 µL of serially diluted auranofin, AU1, or AU5 was added at concentrations ranging from 256 to 0.125 µg/mL. Triton-X at 2% was included as positive control and PBS alone was used as a negative control. The plate was incubated for 1h at 37°C. After incubation, the plate was centrifuged at 1000 g for 5 min. Then, 100 µL of supernatant was transferred to a fresh 96-well microtiter well plate and the OD was measured at 540 nm. The assay was conducted in triplicate and the mean and SD values were reported.

Human primary hepatocytes toxicity. Cryopreserved human primary hepatocytes were purchased from the Cell Resource Core at Massachusetts General Hospital (Boston, MA, USA). Collagen sandwiched configuration was used to culture the thawed cells in 24-well plates. The 24-well plates were coated with rat tail collagen type I (Cell Recourse Core, MGH) and incubated for an hour before 4 x 10^5^ live cells were plated and incubated at 37°C with 5% CO_2_ for 1 h to allow cells to attach to the collagen gel. After incubation, the medium was removed along with dead cells, and the remaining cells received 0.5 mL of hepatocyte culture medium containing Dulbecco’s modified Eagle’s medium (DMEM) media supplemented with 10% FBS, 0.5 U/mL insulin, 14 ng/mL glucagon, 20 ng/mL EGF, 7.5 µg/mL hydrocortisone and 200 U/mL penicillin-streptomycin. The cell cultures were incubated in a humidified, 5% CO_2_ incubator at 37°C. After 24 h, a top layer of collagen gel was poured over the hepatocytes attached to collagen on the bottom of the plate, making a sandwich model. After 1 h, investigational compounds at the designated concentrations were added and incubated for 24 h. Post incubation, hepatocyte WST1 (Roche, Basel, Switzerland) was added and incubated for 4 h. Cell staining was measured by recording the absorbance at 420 nm, measured using a Vmax microplate reader (Molecular Devices, San Jose, CA, USA.), and data were plotted using GraphPad Prism. Cells were tested in triplicate.

### hERG measurement

QPatch HTX system (Sophion Biosciences A/S, Ballerup, Denmark) was used to examine hERG signaling (Cyprotex, Watertown, MA, USA). The system was primed with appropriate extracellular (NaCl, KCl, CaCl2, MgCl2, D-glucose, HEPES, pH 7.4) and intracellular (KCl, MgCl2, EGTA, MgATP, HEPES, pH 7.2) solutions before conducting the study in a 48 well plate (QPlate, Sophion Biosciences A/S, Ballerup, Denmark). All cell suspensions, buffers, and test compound solutions were kept at room temperature during the experiment. HEK293 cells stably transfected with hERG cDNA were washed and positioned into each well (recording chamber) of the QPlate. The QPatch system follows the general principles of conventional whole-cell patch-clamping: a high resistance seal is formed between the patch electrode, and an individual cell, the membrane across the electrode tip is then ruptured, and the whole-cell patch-clamp configuration is established. The standard voltage profile was as follows: step from -80 mV to –50 mV for 200 ms, +20 mV for 4.8 s, step to -50 mV for 5 s, then stepped to the holding potential of -80 mV. The step from -80 mV to the test command (+20 mV) results in an outward current (i.e., current flows out of the cell), and the step from the test command (+20 mV) to -50 mV results in the tail current (the tail current represents deactivation of the current overtime). Compound dilutions were prepared by diluting a DMSO solution (default 10 mM), followed by dilution into an extracellular buffer such that the final concentrations tested were 0.1, 1, and 10 μM (final DMSO concentration 0.1%). Perfusion solutions contained 0.05% Pluronic F-68 and were stored at room temperature for the duration of the experiment each day. The voltage protocol was run and recorded continuously during the experiment. Cells were treated with a vehicle for 3 min (i.e., 0.1% DMSO) followed by three increasing test substance concentrations (0.1, 1, and 10 μM). The test compounds were applied in triplicate at each concentration and tested in at least 2 cells (biological replicates). The standard combined exposure time was 5 min. One vehicle group was run per experiment. In the vehicle group, the vehicle (0.1% DMSO) was dispensed, followed by the addition of positive control (compound E- 4031).

### Compound solubility in DMSO

To test compound solubility, stock solutions were made in triplicate at 50 mg/mL in DMSO. Two-fold serial dilutions of the test compounds were prepared at 50 μL volume in a 96-well assay plate and were kept in the incubator for 1 h. After incubation, the absorbance was measured at 540 nm using a Vmax microplate reader (Molecular Devices, Sunnyvale, CA, USA.) to detect turbidity.

Compound solubility in PBS. Compound solubility was measured in PBS. Briefly, serial dilutions of test compounds were prepared in DMSO at 100x the final concentration. Test compound solutions were diluted 100-fold into PBS in a 96-well plate and mixed. After 2 h, the presence of precipitate was detected by measuring absorbance at 540 nm. An absorbance value greater than ‘mean + 3x standard deviation of the blank’ (after subtracting the background) was considered an indication of precipitates.

### Statistical analysis

GraphPad Prism9.0 was used to graph data. The Dunnel-multiple comparison test was used via One-way ANOVA to see the significance between groups. TrxR enzymatic activity was compared between analogs and DMSO or auranofin and DMSO using Welch’s t-test. Biofilm formation and mature biofilms were compared to untreated biofilms using a Student’s t-test.

## Results

### Subscreen

**Figure 1:**
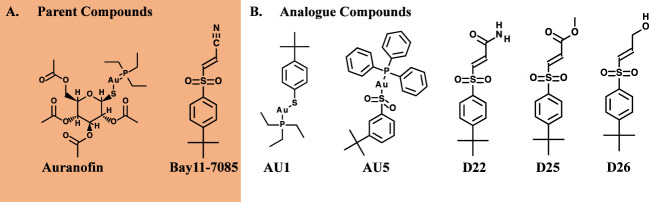
Antibacterial properties of hybrid compounds. **(A)** Parent compound structures. **(B)** Hybrid investigational molecules were synthesized for analysis.

We previously utilized a *C. elegans*-based HTS as an infection model to screen over 80,000 compounds (Harvard University ICCB, Cambridge, MA, USA) that yielded 169 hits (Rajamuthiah et al., 2014; Fuchs et al., 2016; Kim et al., 2018). Among them was auranofin, which has been shown to target *S*. *aureus* TrxR (Harbut et al., 2015). To identify additional TrxR inhibitors, a target-specific subscreen of the 169 hit compounds was completed using DMSO as a negative control and auranofin as a positive control. For this assay, a cloned version of *S*. *aureus* TrxR was used to measure the conversion of DTNB to TNB. TrxR inhibitors would reduce the conversion process. From the screen, Bay11-7085 was defined as a hit based on reduced TrxR enzymatic activity with a 2SD reduction compared to DMSO (**Figure 1A**). Bay11-7085 inhibited TrxR enzymatic activity yielding an OD_420_ of 0.1094 after a 30 min incubation period. In the same time frame, DMSO yielded an OD_420_ of 0.483 ± 0.121 and auranofin had a reduced OD_420_ of 0.295 ± 0.012. The reduction in enzymatic activity in the presence of Bay11-7085 and auranofin demonstrated direct TrxR enzymatic activity inhibition.

### Antimicrobial activity

Based on low MIC values (**Table 1**), Bay11-7085 and auranofin were used as base compound structures to develop analogs. A set of molecules were designed to be hybrids of the hit compounds auranofin and Bay11-7085 (**Figure 1A**) using Glide software from Schrödinger. The process yielded hybrid analogs AU1, AU5, D22, D25, and D26 (**Figure 1B**). These compounds have the S-Au-P moiety from auranofin (AU1) or the sulfonyl olefin from Bay11-7085 (D22, D25, and D26). We explored the hybrid compounds by introducing the sulfone-based S-Au-P moiety for AU5. Among these, AU1 and AU5 were highly active anti-*S*. *aureus* compounds with MICs of 0.125 and 0.25 μg/mL, respectively (**Table 1**). Anti-*S*. *aureus* activity extended beyond the *S*. *aureus* reference strain MW2 and was observed for 30 clinical isolates, ranging from 0.06 to 0.5 μg/mL (**Supplemental Table 1**). The collection included both methicillin-sensitive (MSSA) and resistant (MRSA) isolates.

**Table 1:** Minimal inhibitory concentration of compounds against bacteria.

	*S. aureus* *MW2*	*E. faecium* ATCC E007	*H*. *pylori* 49503	*A. baumannii 17978*	*P. aeruginosa* *PA14*	*K. pneumoniae ATCC 77326*	*Enterobacter (EAE 2625)*
	MIC (μg/mL)	MIC (μg/mL)	MIC (μg/mL)	MIC (μg/mL)	MIC (μg/mL)	MIC (μg/mL)	MIC (μg/mL)
**AU1**	0.125	0.25	0.06	>64	>64	>64	>64
**AU5**	0.25	0.5	1	>64	>64	>64	>64
**Ag analog**	2	2	1	32	64	>64	>64
**Auranofin**	0.25	0.5	0.25	32	>32	>32	>32
**Bay11-7085**	4	32	NT	64	>64	>64	>64
**Vancomycin**	1	1	>64	>64	>64	>64	>64

NT, Not tested.

Neither AU1 nor AU5 exhibited inhibitor activity against Gram-negative bacteria (*Acinetobacter baumannii*, *Pseudomonas aeruginosa*, *Klebsiella pneumonia*, or *Enterobacter* spp.). This was not surprising since most Gram-negative bacteria have a GSH system that also acts as an antioxidant. To test this theory, we evaluated AU1 and AU5 efficacy against *Helicobacter pylori*, a Gram-negative bacterium that lacks GSH. Indeed, *H*. *pylori* were susceptible to both AU1 and AU5 at MICs of 0.06 and 1 μg/mL, respectively (**Table 1**).

### Target inhibition

**Figure 2:**
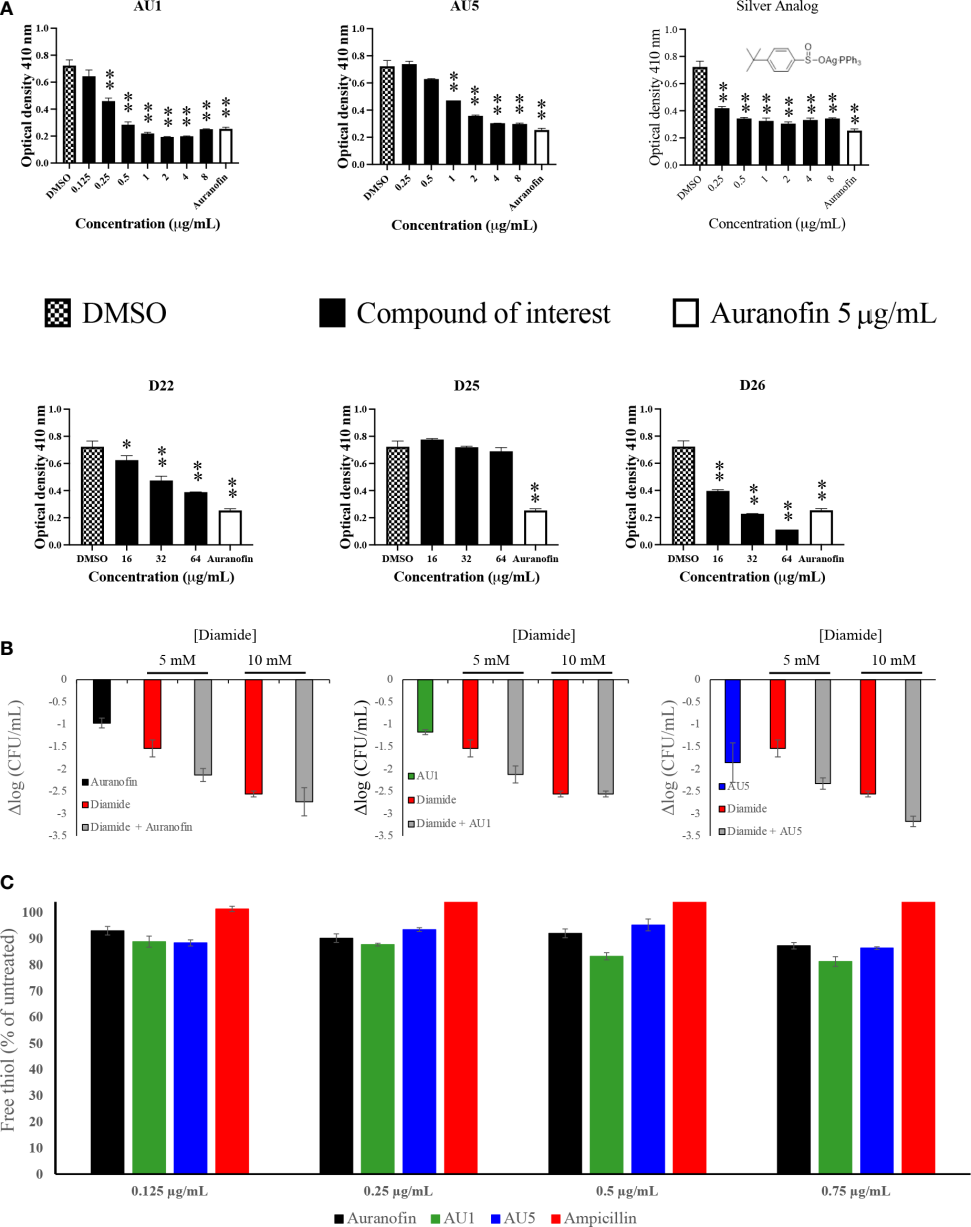
TrxR is inhibited by lead compounds. **(A)** TrxR was evaluated using 40 μL enzyme (0.5 μg/μL) in the reaction mixture for 15 minutes for each of the investigational analogs at serial concentrations. Each concentration was compared to DMSO using Welch’s t-test. Auranofin was included as a positive control. AU1 and AU5 resulted in dose-dependent TrxR inhibition. (* *P*< 0.05; ** *P*< 0.001) **(B)** Combinations of investigational compounds and diamide caused a reduction in *S*. *aureus* CFUs after a 3 h incubation period. *S*. *aureus* cultures were treated with diamide at 5 or 10 mM alone or in combination with AU1, AU5, or auranofin at 0.5 μg/mL. **(C)** TrxR inhibitors reduced intercellular free thiol after a 30 min incubation period. Free thiol was evaluated using a fluorescent thiol assay that compared lysate from treated cells to untreated cells. Ampicillin was included as a negative control.

Among the tested compounds, gold (Au) was a common element present in AU1 and AU5, compared to compounds D22, D25, and D26. To evaluate the significance of gold, we synthesized an analog in which gold was substituted with silver (Ag). Altering the heavy metal component increased the MIC to 2.0 μg/mL, a 16-fold change from a similar molecule (**Figure 2**). Thus, suggesting that gold could play an important role in antimicrobial activity, specifically in TrxR inhibition.

*S*. *aureus* TrxR inhibition was confirmed using an enzymatic assay that measured the conversion of DTNB to TNB. AU1 and AU5 demonstrated dose-dependent inhibitory activity in line with the low MICs and each significantly altered DTNB to TNB demonstrating reduced TrxR enzymatic activity (*P*<0.01) (**Figure 2A**). The IC_50_ was calculated using a nonlinear regression analysis for dose response inhibition and found that AU1 had an IC_50_ of 0.24 μg/mL and AU5 was 1.2 μg/mL. Compounds D22 and D26 demonstrated inhibitory activity but required higher concentrations than AU1, AU5, or auranofin. D25 did not inhibit *S. aureus* TrxR enzymatic activity. To once again, evaluate the significance of gold in the AU1 and AU5 compounds, TrxR inhibitor activity was evaluated using the silver analog. TrxR inhibitory activity was achieved but failed to meet the threshold set by auranofin as a control compound. Further, the tested concentration did not yield a dose-dependent curve as seen with AU1 and AU5.

AU1 and AU5 were investigated further for their impact as TrxR inhibitors. Disruption of the thioredoxin system should lead to ROS accumulation that impedes redox homeostasis. Lead compounds and auranofin were evaluated in combination with diamide, a known thiol-oxidizing agent. Combinations involving AU1, AU5, or auranofin compounds further reduced the *S*. *aureus* growth compared to diamide alone (**Figure 2B**). Millimolar quantities of diamide were able to impact *S*. *aureus* resulting in a 1.5 log CFU reduction at 5 mM diamide and up to 2.6 log CFU reduction at 10mM. When combined with AU1, AU5, or auranofin, the impact of 5 mM diamide was amplified to 2.1 log, 2.3, and 2.1 log CFU reductions, respectively (**Figure 2B**).

Harbut and colleagues previously demonstrated that auranofin inhibition of TrxR caused a decrease in free thiols (Harbut et al., 2015), therefore this idea was explored for AU1 and AU5. By comparing free thiols in lysate from treated cells to untreated cells, a reduction was observed for auranofin as previously described (Harbut et al., 2015). At the tested concentrations, AU1 caused a 19% reduction in free thiol, and AU5 caused a 16% reduction in the 30-minute test period (**Figure 2C**). By contrast, treatment with ampicillin had no impact on free thiols.

To further evaluate the inhibitory activities of AU1 and AU5 which incorporate the S-Au-P moiety, the building blocks and possible decomposition products of AU1 and AU5 were tested for antibacterial activity. These include the 4-terbuyl benzenethiol, 4-terbuyl benzenethiol sulfinic acid, and triethyl- and triphenyl phosphine oxides. As shown in **Supplemental Figure 2**, none of these compounds yielded a MIC lower than 128 μg/mL.

### Antimicrobial kinetics

**Figure 3:**
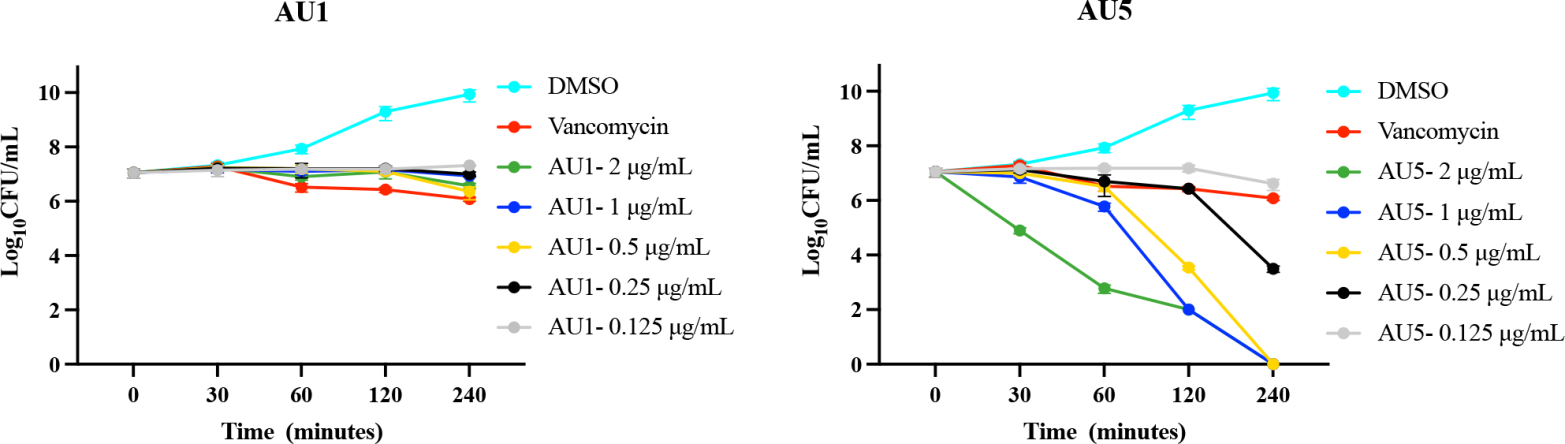
Antimicrobial kinetics. Log-phase MRSA-MW2 cells were treated with various concentrations of investigational compounds and aliquots of the cells were drawn at regular intervals. Viable cells were quantified by culturing and enumerating the CFU. AU1 was bacteriostatic and AU5 resulted in reduced growth, suggesting it is bacteriocidal.

Antimicrobial and target assessment reveal AU1 and AU5 as lead anti-*S*. *aureus* compounds. Time-to-kill kinetics were used to evaluate the bactericidal versus bacteriostatic nature of the compounds. According to Lee and Burgess (2013), any compound that reduces the cell population by 3-log_10_-CFU/mL compared to the initial inoculum concentration is considered bactericidal (Lee and Burgess, 2013). By this definition, AU5 was bactericidal and AU1 was bacteriostatic (**Figure 3**). Although AU1 demonstrated a low MIC (0.125 μg/mL), the killing kinetics reveals static growth over a 4 h test period.

### Biofilm inhibition

**Figure 4:**
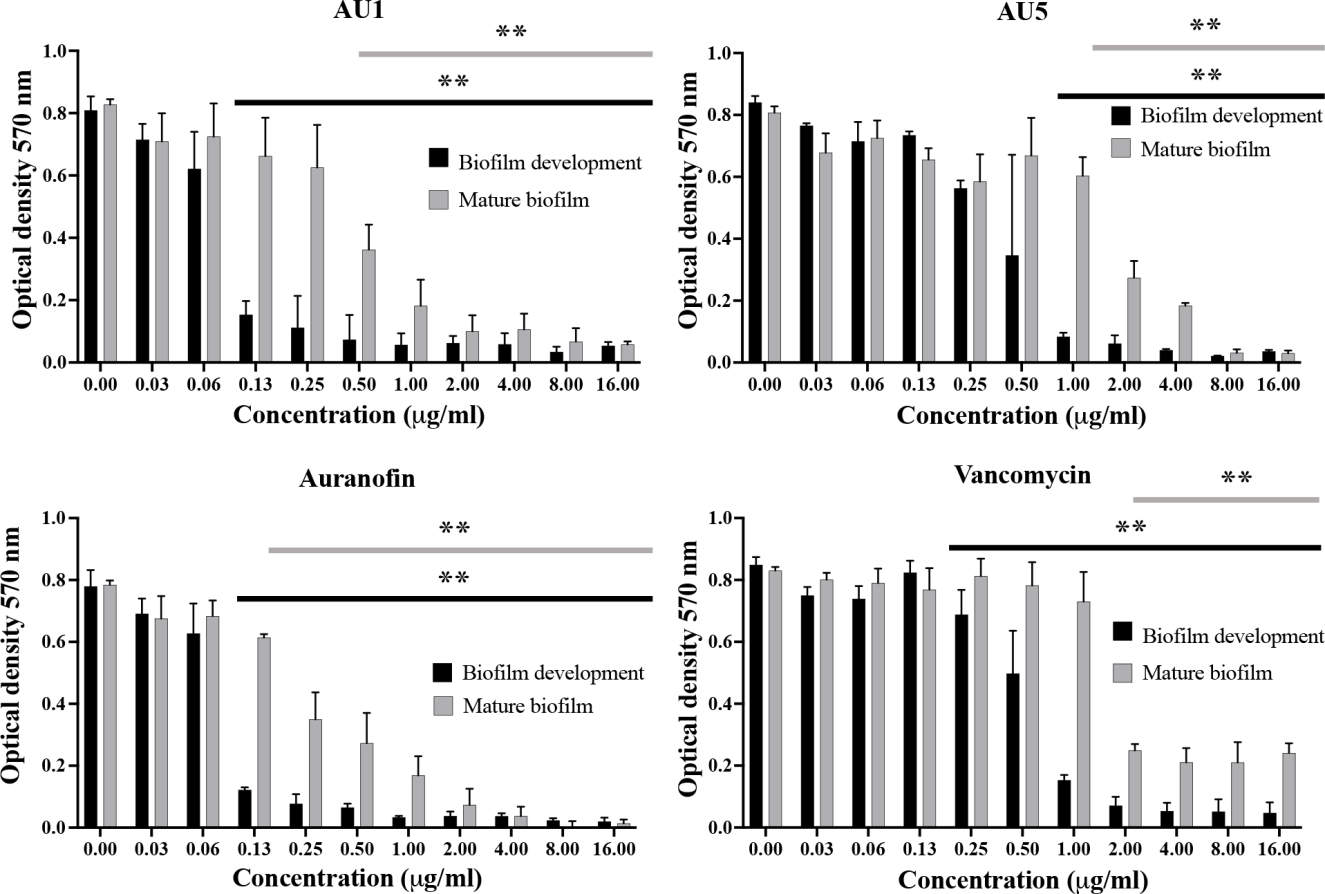
*S*. *aureus* biofilm was inhibited by lead AU1 and AU5 compounds. Biofilm formation and biofilm accumulation from mature biofilms were measured to evaluate the impact of AU1 and AU5 compound exposure. Both investigational compounds were able to disrupt *S*. *aureus* biofilm at low concentrations, comparable to the control compound auranofin and superior to vancomycin. ** (*P*<0.01) indicated significance for a series of concentrations that reduce biofilm formation or mature biofilms.

Auranofin is known to inhibit the process of biofilm formation and also disrupt preformed biofilms made by *S*. *aureus* or other microbial organisms, such as *Candida albicans* (Siles et al., 2013; Torres et al., 2016; Liu et al., 2019; She et al., 2020). Evaluation of auranofin as a control compound demonstrated inhibition of biofilm formation and disruption of mature biofilms in support of previous findings. Auranofin was able to inhibit biofilm formation at concentrations as low as 0.125 μg/mL (*P*< 0.01). Mature *S*. *aureus* biofilm was also significantly reduced at 0.125 μg/mL (*P*< 0.01) (**Figure 4**). By contrast, vancomycin, a standard-of-care antibiotic, reduced biofilm formation at 1 μg/mL (*P*<0.01), and mature biofilm was significantly reduced compared to the control at 2 μg/mL (*P*<0.01), but does not completely inhibit mature biofilms. AU1 inhibited biofilm formation at 0.125 μg/mL (*P*<0.01), and mature biofilms at 0.5 μg/mL (*P*<0.01), concentrations in line with control compound auranofin. AU5 was also effective at inhibiting biofilms but not to the same degree as AU1. Biofilm formation was reduced at 1 μg/mL (*P*<0.01), and mature biofilms were significantly reduced at 1 μg/mL. It was significant that AU1 was effective at disrupting *S*. *aureus* biofilms, a state that is normally difficult to impact for many antibiotics.

### Cytotoxicity

**Figure 5:**
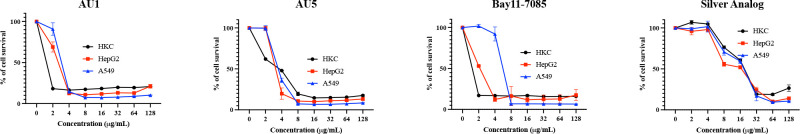
Hybrid molecules were assessed for cytotoxic properties in cell lines. Cytotoxicity of AU1 and AU5 were examined for HKC, HepG2, and A549 cells. Cell survival was also evaluated for Bay11-7085 and a silver analog as references. Each of the cell lines was measured for survival when exposed to increasing concentrations of the investigational compounds. Mean and SD are reported in the graphs.

The cytotoxicity of AU1 and AU5 was tested *in vitro* with immortalized and primary cells, gauging the liability of lead formulations. Cell lines used to evaluate compound toxicity included: HKC, a kidney cell line, HepG2, a liver cell line, and A549, a lung carcinoma cell line. For AU1, HKC LD_50_ was <2 μg/mL. Using HepG2 as a means to evaluate toxicity, the LD_50_ for AU1 was approximately 3 μg/mL, and for A549 the LD_50_ was approximately 4 μg/mL (**Figure 5**). Thus, suggesting that AU1 was toxic to the tested cancer cell lines at 16 to 32X the MIC. The LD_50_ of AU5 for HKC, HepG2, and A549 cells was approximately 4 μg/mL (16X the MIC). A compound analog that replaced gold with silver was tested and a reduction in toxicity was observed that extended the LD_50_ from 2 – 4 μg/mL to approximately 16 μg/mL.

BAY 11-7085, the parent structure from which AU1 and AU5 were derived, served as another non-gold analog and exhibited toxicity between 2 and 8 μg/mL for the three cell lines tested. Therefore, it may or may not be the gold component strictly contributing to cell toxicity, the issue could be related to the thioredoxin reductase target itself. TrxR is known to be elevated in some cancer cell types and inhibition by auranofin or shikonin leads to apoptosis (Duan et al., 2014; Hwang-bo et al., 2017). While immortalized cell lines are one way of testing toxicity in early-stage drug development it’s not the only option.

**Figure 6:**
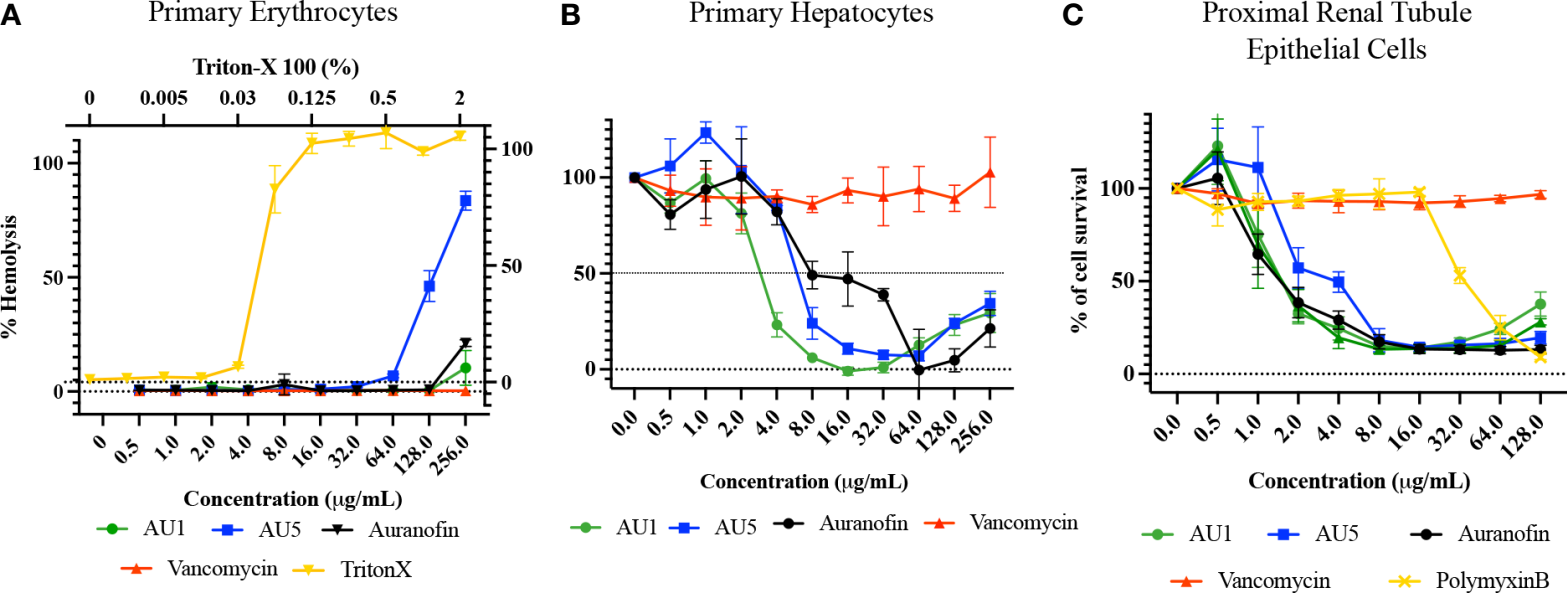
Lead molecules were evaluated for cytotoxic liability using primary cells. **(A)** Human primary erythrocytes were assessed for hemolysis caused by AU1 and AU5. Standard of care antibiotic vancomycin was included as a reference. Triton X was included as a positive hemolysis control. **(B)** Primary human hepatocyte survival was assessed with various concentrations of AU1 and AU5. Cell survival was compared to auranofin as a tool reference compound and vancomycin as a standard-of-care antibiotic. **(C)** Proximal renal tubule epithelial cells were assessed for AU1 and AU5 sensitivity using auranofin as a control compound and vancomycin and polymyxin B as reference compounds in the assay.

Human primary erythrocytes, hepatocytes, and renal proximal tubule cells were assessed for sensitivity to AU1 and AU5, measuring hemolysis and cell survival, respectively. Erythrocytes were selected because they are commonly exposed upon compound injection. As the body detoxifies and excretes compounds, they are exposed to the liver and kidney, making these additional cell types significant for testing cytotoxicity. AU1 did not cause hemolysis within the tested range indicating hemolysis LD_50_ exceeded the MIC by at least 2000X (**Figure 6A**). However, AU5 caused hemolysis to erythrocytes reaching an LD_50_ at <128 μg/mL, approximately 256X the MIC. Erythrocytes appear not to be adversely affected by AU1 and hemolysis caused by AU5 is at concentrations that greatly exceed the MIC. For primary hepatocytes, an LD_50_ was reached at approximately 3 μg/mL for AU1 and 6 μg/mL for AU5, 24X the MIC for each compound (**Figure 6B**). By comparison, the reference compound auranofin reached an LD_50_ at approximately 8 μg/mL which is 32X the MIC. Although hepatocyte cell death was measured within the tested range, LD_50_ occurred at concentrations that exceeded the effective MICs. Renal proximal tubule epithelial cells were tested and resulted in an LD_50_ of 1.5 and 2 μg/mL for AU1 and AU5, respectively (**Figure 6C**). The LD_50_ for AU1 was 12X MIC compared to the control compound auranofin, which was 6X MIC. Vancomycin was included as a reference in the assay and did not impact renal proximal tubule epithelial cells at the tested concentrations. Nephrotoxicity can result from polymyxin B (De Fátima Fernandes Vattimo et al., 2016) which was included as a reference control.

### hERG assessment

**Figure 7:**
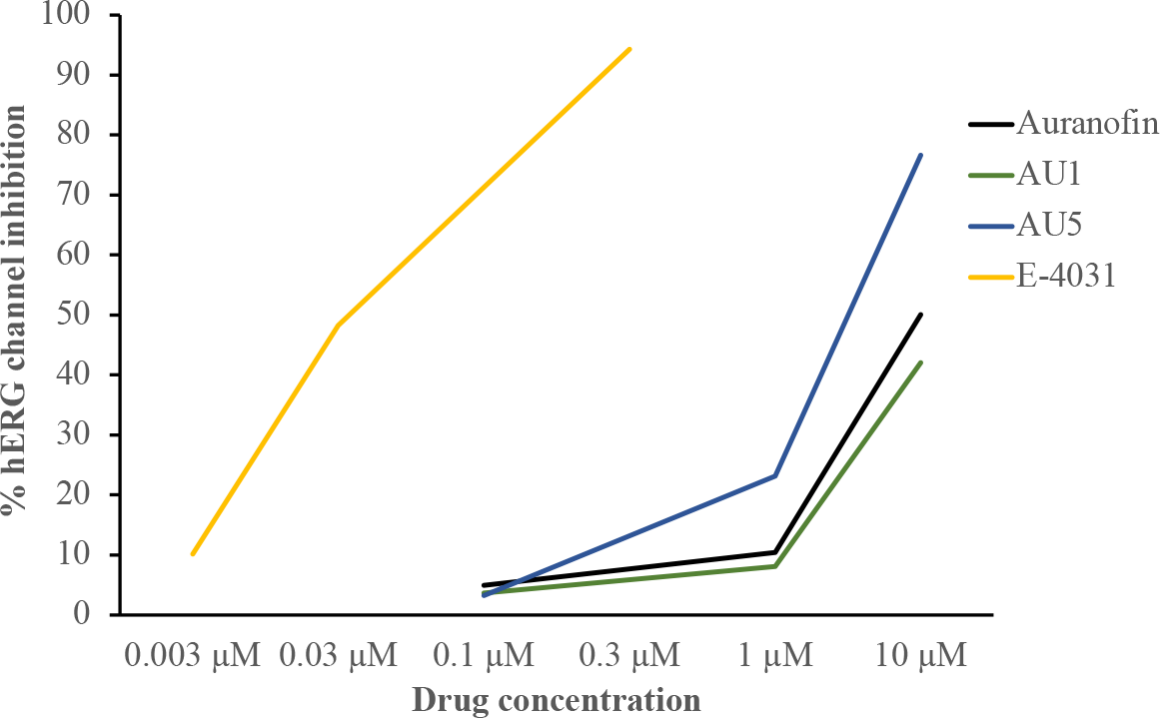
AU1 and AU5 do not inhibit the hERG channel. Compounds were evaluated for hERG inhibition. Auranofin generated an IC_50_ value of 9.68 μM, and AU1 had an IC_50_ greater than the highest tested value of 10 μM. AU5 exhibited an IC_50 = _3.16 μM. The positive control to demonstrate hERG inhibition was E-4031, which yielded an IC_50 = _0.03 μM.

Ether-á-go-go Related Gene (hERG) encodes for the alpha subunit of a potassium ion channel that contributes to the electrical current facilitating the heart beating (Garrido et al., 2020). Investigational compounds should exceed an IC_50_ value of 1 μM to prevent toxic events in the heart. The IC_50_ value for AU1 exceeded 10 μM and AU5 was 3.16 μM, indicating both compounds reached the necessary threshold (**Figure 7**). E-4031 was used as a control hERG inhibitor and auranofin was included as the reference control compound.

### Solubility

The solubilities of AU1 and AU5 were tested in PBS and DMSO. Using PBS as a solvent, both AU1 and AU5 solubilities were 3.13 μM (**Supplemental Figure 3A**), which equates to 1.49 μg/mL and 2.03 μg/mL of the compounds, respectively. With DMSO as the solvent, AU5 began to exhibit reduced solubility at 6.5 μg/mL but AU1 remained soluble up to 50 μg/mL (**Supplemental Figure 3B**). Overall, solubility was improved using DMSO as the solvent. When testing solubilities in both solvents, high and low-solubility compounds, verapamil and reserpine, were included as references. AU1 and AU5 demonstrated limited solubility in PBS but this could be potentially improved through the addition of hydrophilic side chains in next-generation analogs.

## Discussion

Drug-resistant or tolerant strains in the form of MRSA, vancomycin-resistant *S*. *aureus* (VRSA), and vancomycin-intermediate *S*. *aureus* (VISA) continue to cause infections that are challenging to treat with the current antimicrobial drug arsenal (Cuddy, 2008). The ongoing use of salvage therapies such as daptomycin strains the limited drug arsenal and daptomycin-resistant bacteria continue to emerge through mutations that impact proteins of the lipid metabolic pathways and two-component sensors involved in cell envelope homeostasis (Guiqing et al., 2018; Nguyen et al., 2022). Thus, drug resistant microbes continue to emerge and demonstrate the limitations of the current antimicrobial arsenal that focus on a limited number of bacterial targets. With the continued evolution of drug resistance, new therapeutics and targets are needed. TrxR could provide a valuable new target and AU1 and AU5 along with published studies showing the antimicrobial efficacy of auranofin demonstrate this possibility. The lead S-Au-P scaffolded compounds exhibit antimicrobial activity by targeting TrxR, a Gram-positive essential system required for redox homeostasis.

In Gram-negative bacteria, the thioredoxin system is backed up by the GSH system to maintain oxidative homeostasis. However, for Gram-positive bacteria and some Gram-negative bacteria, the systems are not redundant, leaving the thioredoxin system to serve the essential task of defending against oxidative stresses through disulfide reductase activity (Baker et al., 2001; Uziel et al., 2004). Auranofin, which inhibits the growth of several bacteria, and BAY 11-7085 offer proof of concept and provide insights to advancing new small molecules that utilize TrxR as a target (Rhodes et al., 1992; Bonilla et al., 2008; Angelucci et al., 2009; Jackson-rosario et al., 2009; Jackson-Rosario and Self, 2009; Debnath et al., 2012; Tejman-Yarden et al., 2013; Cassetta et al., 2014; Harbut et al., 2015; Fuchs et al., 2016; Tharmalingam et al., 2019). Using auranofin and Bay11-7085 as control compounds, AU1 and AU5 were designed and were found to exhibit antimicrobial activity against *S*. *aureus* and *E*. *faecium*. Insightfully, these lead molecules also inhibit *H*. *pylori*, an exception within the Gram-negative category that happens to be GSH-independent, meaning that redox homeostasis is dependent on the Trx system (Jonkers et al., 1999; Aydin et al., 2000; Koçkar et al., 2001).

*H*. *pylori* susceptibility to AU1 and AU5 contrasted with the lack of susceptibility by other Gram-negative bacteria reiterates that TrxR is a likely compound target. Using a TrxR enzymatic assay, control compounds and leads AU1 and AU5 were found to diminish TrxR redox activity. The target was not impacted by standard-of-care antibiotics. Although pharmacokinetic analysis of AU1 and AU5 is still needed, TrxR inhibitor efficacy will be based on reaching serum concentrations at levels that reach *S*. *aureus* MICs.

Gold appeared to be important to the S-Au-P scaffold to achieve antimicrobial efficacy through TrxR inhibition. Although not effective *S*. *aureus* inhibitors, the compounds D22, D25, and D26 provided the opportunity to explore an alternate chemical moiety from the parent compounds. Failure of the D series to achieve the same low MIC concentrations against *S*. *aureus* that were found in the AU series suggests the significance of the S-Au-P portion of the compounds. Gold-containing compounds have had limited therapeutic use, rheumatoid arthritis being one, the primary agents being auranofin and aurothioglucose (Kean et al., 1997). Limited use is attributed in part to nephrotoxicity and chemical stability (Nobili et al., 2010). Proteinuria was reported in 5% of auranofin-treated patients and hypothesized to be a result of gold or gold complexes (Kean et al., 1997). However, short-term use as an antimicrobial agent may not elicit the same adverse events as daily, long-term dosages for a chronic condition. Despite the limitation of gold-based therapeutics, usage continues in cases where more conventional drugs fail and alternative therapeutics are needed.

Gold has been noted for its antimicrobial properties and this may be conveyed through the TrxR target. When gold was replaced by silver in this study, TrxR inhibitory activity was reduced. Comparably, the removal of gold from auranofin analogs by Aguinagalde et al. caused a loss of antimicrobial activity against *Streptococcus pneumoniae*, suggesting gold aids in asserting antimicrobial properties to auranofin (Aguinagalde et al., 2015). Initial elements of the structure-activity relationship groundwork were laid by Angelucci and colleagues who found that auranofin releases its gold atom to facilitate Trx inhibition (Angelucci et al., 2009). Although the findings are debatable based on results presented by Parsonage et al. who demonstrated that the interactive molecular mechanism of auranofin with *Entamoeba histolytica* TrxR caused a modification of Cys^286^, possibly a non-catalytic site, it is still likely that auranofin impacts TrxR function (Parsonage et al., 2016). When gold-containing N-heterocyclic carbene ligands containing benzimidazole were created by Özdemir and colleagues, the synthesized molecules exhibited lower MICs against Gram-positive bacteria than Gram-negative (Ozdemir et al., 2010). Based on the findings that auranofin can inhibit TrxR to yield antimicrobial activity, we introduce a new class of TrxR inhibitors through the synthesis of AU1 and AU5.

The recalcitrant nature of the biofilm matrixes that form a physical barrier reduces antimicrobial inhibitory activity by excluding even standard-of-care medications (Lister and Horswill, 2014; Suresh et al., 2019; Idrees et al., 2021). Auranofin is noted for its efficacy against both planktonic and biofilm states, impacting biofilm formation and mature biofilms (Torres et al., 2016). In a study by Aguinagalde and colleagues (2015), auranofin significantly reduced *S*. *aureus* biofilm accumulation in a murine polypropylene mesh-associated biofilm model (Aguinagalde et al., 2015). Inhibitory activity extends to dual pathogen biofilms created by *S*. *aureus* and *C. albicans* (She et al., 2020). The newly created AU1 and AU5 compounds with similar S-Au-P scaffolds were able to reduce biofilm formation and mature biofilms, indicating efficacy at inhibiting *S*. *aureus* growth in multiple physical states.

Although auranofin and the lead compounds AU1 and AU5 demonstrate significant antimicrobial properties, the potential for toxic effects cannot be ignored. When evaluated in immortalized cell lines, AU1 and AU5 demonstrated toxicity at 16 to 32X MIC concentrations, but inhibition exceeded antimicrobial MICs. TrxR can be elevated in some cancer cell lines, which may have also impacted susceptibility to the test compounds (Duan et al., 2014; Hwang-bo et al., 2017). Replacing gold with silver reduced the toxic liability so indeed gold may be an influence. According to Kean et al. there is a lack of targeted clinical trials that study the cytotoxic effects of gold (Kean and Kean, 2008). However, the most prominent side effect of injectable gold containing compounds for the treatment of chronic rheumatoid arthritis is skin rash.

Cytotoxicity was evaluated in primary human cell types, providing a more resolute characterization of the compounds. Cytotoxicity against human erythrocytes exceeded AU1 and AU5 concentrations by more than 2,000 and 256X, respectively. Using primary human hepatocytes to gauge the impact of AU1 and AU5, LD_50_ concentrations were 24X the individual MICs, slightly lower than the control compound, auranofin, which is already an FDA-approved therapeutic for treating chronic rheumatoid arthritis and inhibited primary hepatocytes at 32X the MIC. AU1 and AU5 were more toxic to primary renal proximal tubule epithelial cells at 12X and 8X the MICs, respectively. Thus, AU1 and AU5 pose a modest amount of toxic liability to hepatocytes and more directly to renal cells, but there is potential to improve the compound structure to limit the liability. Higher toxicity in the immortalized cell lines was not surprising since some cancer cells have elevated levels of TrxR to prevent ROS accumulation in support of accelerated growth rates (Duan et al., 2014; Hwang-bo et al., 2017). However, the toxicity was not as high in primary cell types. Greater tolerability in normal cell types might be associated with mammals having multiple copies of orthologous TrxR in addition to an orthologous GSH system. Humans have cytosolic and separate mitochondrial thioredoxin to facilitate redox regulation (Lu and Holmgren, 2014). This dual system with multiple alleles can then tolerate the small concentration of TrxR inhibitors that impacts Gram-positive bacteria with a single allele and lack the GSH antioxidant system. The likely significance of the lone TrxR system is illustrated by the lack of TrxR inhibitor sensitivity in Gram-negative bacteria that do have a GSH system.

Within the drug development process, one of the most frequent adverse side effects is cardiac arrhythmia due to the off-target effects of hERG. Importantly, the investigational lead compounds AU1 and AU5 do not adversely affect hERG, suggesting there is a low likelihood of arrhythmia from disruption to the heart potassium channel. Overall, the evaluation of AU1 and AU5 with both cell lines and primary cells demonstrates that mammalian cell disruption occurs at concentrations that exceed those of bacterial inhibition, and, with future modification through pharmacokinetic analysis, a safe compound could potentially be achieved. Further, AU1 and AU5, or any future derivative, will most likely be reserved for drug-resistant microbes that fail to be resolved by standard-of-care antibiotics, and the low efficacious inhibitory concentrations may not require high dosing when advanced to animal models. Further, short-term use would be likely as antimicrobial therapies.

In conclusion, AU1 and AU5 S-Au-P scaffolded compounds exhibited effective antimicrobial activity. Using *S*. *aureus* as a model Gram-positive bacterium, antimicrobial activity was conserved among clinical isolates and laboratory reference strains. The same anti-*S*. *aureus* efficacious nature against clinical and reference strains was observed with auranofin, which previously inhibited more than 500 isolates (Tharmalingam et al., 2019). Both AU1 and AU5 appear to inhibit TrxR, demonstrating the importance of a functional thioredoxin system to Gram-positive and GSH-independent bacteria. The functional inhibition provided by the AU series of compounds versus the D series (D22, D25, and D26) suggests that gold could provide an affinity for the TrxR docking target that can be explored further. Toxicity levels for the lead compounds (AU1 and AU5) exceed bacterial inhibitory concentrations. The effective nature of the compounds at sub-toxic concentrations suggests that TrxR presents a valuable antimicrobial target to impact drug-resistant Gram-positive bacteria.

## Data availability statement

The raw data supporting the conclusions of this article will be made available by the authors, without undue reservation.

## Author contributions

Experiments were designed and executed by NT and LF. SX, BR, and MX designed and synthesized compounds. EM, MX, and BF designed experiments and directed research. NT and BF wrote the manuscript. All authors analyzed the results, revised the manuscript, and approved the final version.

## Supplementary material

Supplementary Figure 1 TrxR target-specific screen. **(A)** The thioredoxin system provides redox homeostasis by reducing oxidative stressors via the transfer of electrons from TrxR to Trx. **(B)** A standard curve of *S. aureus* TrxR enzyme reduction of DTNB to TNB with and without TrxR inhibitor. Incubation periods of 10 minutes or greater achieved significance (*P*<0.05) when comparing activity with and without the inhibitor. **(C)** Evaluation of *S*. *aureus* TrxR enzymatic activity and inhibition. Auranofin reduced TrxR activity (*P*<0.01) **(D)** Standard antibiotics and anti-*S*. *aureus* compounds were compared to auranofin to gauge TrxR specific inhibitory activity. **(E)** Subscreen of anti-*S*. *aureus* compounds to find TrxR inhibitors. The dotted line represents 2SD reduction from DMSO negative control, representing significant TrxR inhibition. Bay11-7085 is marked in red and circled.

Supplementary Figure 2 Lack of antibacterial properties from lead compound components **(A)**. The MIC was tested for each of the components used to synthesize lead AU compounds. **(B)** Lead compound compositional chemicals were examined for TrxR enzyme inhibition. The components were compared to TrxR inhibition from 5μg/mL auranofin.

Supplementary Figure 3 AU1 and AU5 solubility. Solubility of investigational compounds was tested in PBS and DMSO. **(A)** AU1 and AU5 demonstrated equal solubility in PBS. **(B)** Within DMSO as a solvent, AU1 was more soluble than AU5.

Supplemental Table 1 MIC against clinical isolates of *S. aureus*. Isolates were tested as 2 biological replicates.

Supplemental Materials and Methods The D series of compounds were examined using NMR spectra. For D22, ^1^H NMR (400 MHz, CHLOROFORM-d) δ = 7.91 - 7.75 (m, 2H), 7.64 - 7.53 (m, 2H), 7.42 - 7.23 (m, 1H), 7.11 - 6.86 (m, 1H), 5.97 - 5.59 (brs, 2H), 1.38 (s, 9H). For D25, ^1^H NMR (400 MHz, CHLOROFORM-d) δ = 7.97 - 7.74 (m, 2H), 7.62 - 7.46 (m, 2H), 6.64 - 6.38 (m, 2H), 3.90 (s, 3H), 1.35 (s, 9H). For D26, ^1^H NMR (400 MHz, CHLOROFORM-d) δ = 7.93 - 7.78 (m, 2H), 7.61 - 7.51 (m, 2H), 6.43 (td, *J* = 5.6, 11.3 Hz, 1H), 6.32 - 6.24 (m, 1H), 4.78 - 4.69 (m, 2H), 2.38 - 2.21 (m, 1H), 1.38 (m, 9H).
