# Supplementary material for: Gold complex compounds that inhibit drug-resistant Staphylococcus aureus by targeting thioredoxin reductase
Source: Front Antibiot. 2023 Aug 21;2:1179354. doi: 10.3389/frabi.2023.1179354 (PMC11732138; doi:10.3389/frabi.2023.1179354)
Supplement: Supplementary Figure 1 — TrxR target-specific screen. (A) The thioredoxin system provides redox homeostasis by reducing oxidative stressors via the transfer of electrons from TrxR to Trx. (B) A standard curve of S. aureus TrxR enzyme reduction of DTNB to TNB with and without TrxR inhibitor. Incubation periods of 10 minutes or greater achieved significance (P<0.05) when comparing activity with and without the inhibitor. (C) Evaluation of S. aureus TrxR enzymatic activity and inhibition. Auranofin reduced TrxR activity (P<0.01) (D) Standard antibiotics and anti-S. aureus compounds were compared to auranofin to gauge TrxR specific inhibitory activity. (E) Subscreen of anti-S. aureus compounds to find TrxR inhibitors. The dotted line represents 2SD reduction from DMSO negative control, representing significant TrxR inhibition. Bay11-7085 is marked in red and circled. [file DataSheet_1.pdf]

Supplemental Figure Legends

Supplemental Figure 1: TrxR target-specific screen. A. The thioredoxin system provides redox homeostasis by reducing oxidative stressors via the transfer of electrons from TrxR to Trx. **B.** A standard curve of *S. aureus* TrxR enzyme reduction of DTNB to TNB with and without TrxR inhibitor. Incubation periods of 10 minutes or greater achieved significance ($P<0.05$) when comparing activity with and without the inhibitor. **C.** Evaluation of *S. aureus* TrxR enzymatic activity and inhibition. Auranofin reduced TrxR activity ($P<0.01$) **D.** Standard antibiotics and anti-*S. aureus* compounds were compared to auranofin to gauge TrxR specific inhibitory activity. **E.** Subscreen of anti-*S. aureus* compounds to find TrxR inhibitors. The dotted line represents 2SD reduction from DMSO negative control, representing significant TrxR inhibition. Bay11-7085 is marked in red and circled.

Supplemental Figure 2: Lack of antibacterial properties from lead compound components A. The MIC was tested for each of the components used to synthesize lead AU compounds. **B.** Lead compound compositional chemicals were examined for TrxR enzyme inhibition. The components were compared to TrxR inhibition from 5μg/mL auranofin.

Supplemental Figure 3. AU1 and AU5 solubility. Solubility of investigational compounds was tested in PBS and DMSO. **A.** AU1 and AU5 demonstrated equal solubility in PBS. **B.** Within DMSO as a solvent, AU1 was more soluble than AU5.

Supplemental figure 1

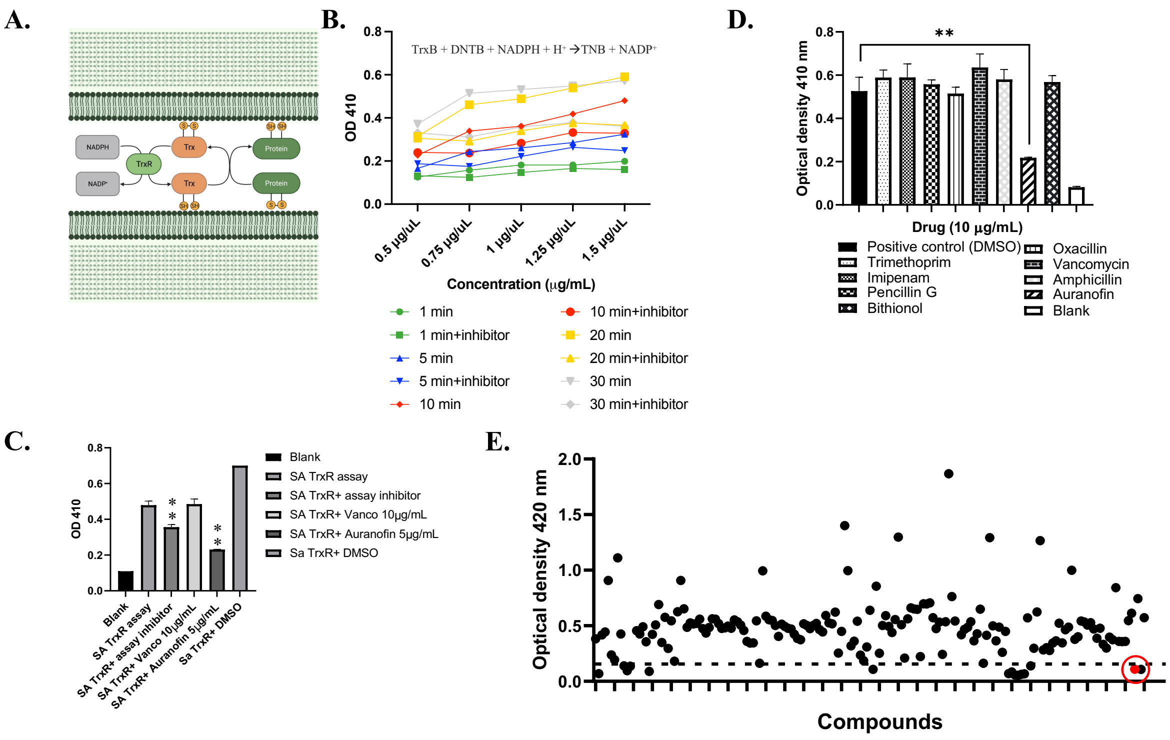

Supplemental figure 2

A.

Component antimicrobial activity

Name	CAS	MIC against *S. aureus* MW2 (µg/mL)
C1-4-tert-butylbenzenethiol	2396-68-1	>128
C2-4-tert-butylbenzenesulfinic acid	88576-64-1	>128
C3-triethylphosphine oxide	597-50-2	>128
C4-triphenylposphine oxide	791-28-6	>128

B.

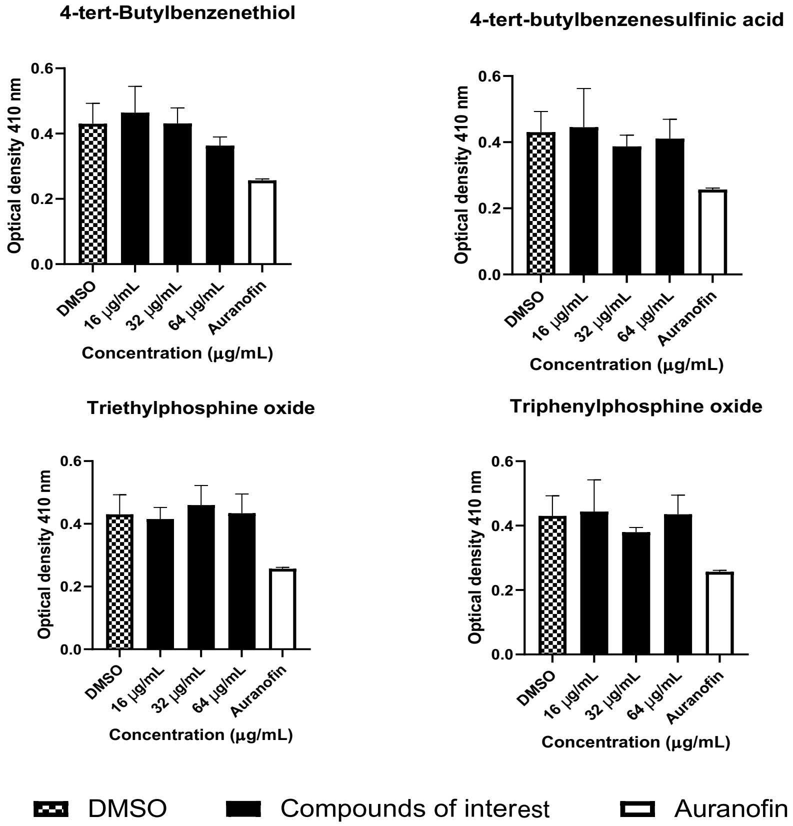

Supplemental Figure 3

A.

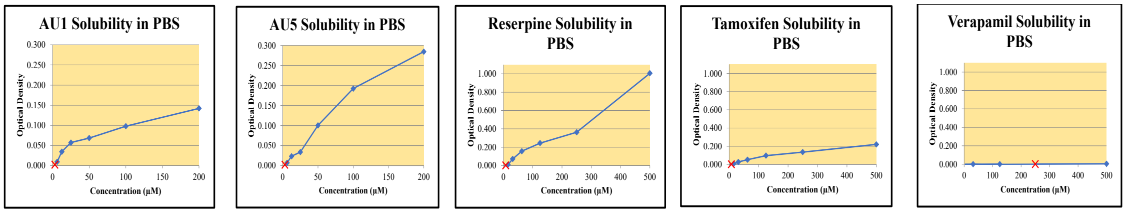

Test Article	Buffer	Solubility Limit (μM)[a] 2 Hour	Comment
**Reserpine**	PBS	7.81	Low solubility control
**Tamoxifen**	PBS	7.81	
**Verapamil**	PBS	250	High solubility control
**AU1**	PBS	3.13	
**AU5**	PBS	3.13	

[a] Solubility limit is highest concentration with no detectable precipitate.

B.

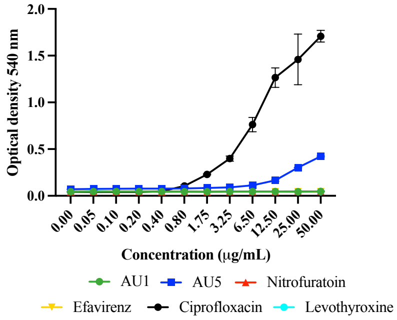

Supplemental Materials and Methods. The D series of compounds were examined using NMR spectra. For D22, ^{1}H NMR (400 MHz, CHLOROFORM-d) δ = 7.91 - 7.75 (m, 2H), 7.64 - 7.53 (m, 2H), 7.42 - 7.23 (m, 1H), 7.11 - 6.86 (m, 1H), 5.97 - 5.59 (brs, 2H), 1.38 (s, 9H). For D25, ^{1}H NMR (400 MHz, CHLOROFORM-d) δ = 7.97 - 7.74 (m, 2H), 7.62 - 7.46 (m, 2H), 6.64 - 6.38 (m, 2H), 3.90 (s, 3H), 1.35 (s, 9H). For D26, ^{1}H NMR (400 MHz, CHLOROFORM-d) δ = 7.93 - 7.78 (m, 2H), 7.61 - 7.51 (m, 2H), 6.43 (td, *J* = 5.6, 11.3 Hz, 1H), 6.32 - 6.24 (m, 1H), 4.78 - 4.69 (m, 2H), 2.38 - 2.21 (m, 1H), 1.38 (m, 9H).
