# Supplementary material for: Gold complex compounds that inhibit drug-resistant Staphylococcus aureus by targeting thioredoxin reductase
Source: Front Antibiot. 2023 Aug 21;2:1179354. doi: 10.3389/frabi.2023.1179354 (PMC11732138; doi:10.3389/frabi.2023.1179354)
Supplement: Supplemental Table 1 — MIC against clinical isolates of S. aureus. Isolates were tested as 2 biological replicates. [file Table_1.pdf]

Supplemental Table 1. MIC against clinical isolates of *S. aureus*

	MIC (µg/mL)			
**Isolate**	**AU1**	**AU5**	**Vancomycin**	**Oxacillin**
BFSA12	0.125	0.25	1	0.5
BFSA13	0.125	0.5	1	1
BFSA15	0.125	0.25	1	0.25
BFSA16	0.125	0.5	1	0.25
BFSA17	0.125	0.25	1	0.125
BFSA18	0.25	0.5	2	0.5
BFSA19	0.125	0.5	1	0.5
BFSA20	0.25	0.5	1	0.5
BFSA21	0.125	0.5	2	0.5
BFSA22	0.125	0.5	2	1
BFSA24	0.125	0.25	1	0.5
BFSA26	0.125	0.25	2	0.25
BFSA29	0.125	0.25	1	>8
BFSA30	0.125	0.25	1	>8
BFSA31	0.125	0.5	2	>8
BFSA32	0.125	0.25	2	>8
BFSA33	0.125	0.25	2	>8
BFSA34	0.125	0.5	1	>8
BFSA35	0.125	0.25	1	>8
BFSA36	0.125	0.5	2	>8
BFSA37	0.06	0.125	1	0.25
BFSA38	0.125	0.25	1	0.25
BFSA39	0.125	0.25	1	0.25
BFSA148	0.125	0.25	1	>8
BFSA149	0.125	0.25	2	>8
BFSA150	0.125	0.5	1	>8
BFSA151	0.125	0.25	1	>8
BFSA152	0.125	0.5	2	>8
BFSA153	0.125	0.25	2	>8
BFSA155	0.125	0.5	1	>8

Isolates were tested as 2 biological replicates.
